# Persistent microvascular obstruction-like lesion after ventricular tachycardia ablation detected by novel dark-blood late gadolinium enhancement

**DOI:** 10.1259/bjrcr.20210124

**Published:** 2022-02-02

**Authors:** Lobke L Pype, Robert J Holtackers, Bernard P Paelinck, Thalia Bekelaar, Hein Heidbuchel, Caroline M Van De Heyning

**Affiliations:** 1 Department of Cardiology, Antwerp University Hospital Drie Eikenstraat, Antwerp (Edegem), Belgium; 2 Research group Cardiovascular Diseases, GENCOR, Antwerp University Universiteitsplein, Antwerp, Belgium; 3 Department of Radiology & Nuclear Medicine, Maastricht University Medical Centre P. Debyelaan, Maastricht, the Netherlands; 4 Cardiovascular Research Institute Maastricht (CARIM), Maastricht University, Duboisdomein, Maastricht, the Netherlands; 5 Department of Cardiac Surgery, Antwerp University Hospital, Drie Eikenstraat, Antwerp (Edegem), Belgium

## Abstract

Microvascular obstruction is a transient phenomenon of “no reflow” after myocardial infarction or radiofrequency ablation, diagnosed using late gadolinium enhancement cardiac MRI. We present a patient with a persistent microvascular obstruction-like lesion following radiofrequency ventricular tachycardia ablation post-myocardial infarction, which was best characterized by a novel dark-blood late gadolinium enhancement technique.

## Introduction

Microvascular obstruction (MVO), also known as “no reflow”, is a well-characterized condition that generally occurs after myocardial infarction or radiofrequency ablation. It is a transient phenomenon, presumably caused by damage to the myocardial microvasculature, and it usually disappears after a few days to weeks. MVO can be diagnosed using cardiac magnetic resonance (CMR) with late gadolinium enhancement (LGE), where it appears as a dark core within the hyperenhanced area of a myocardial scar. Here, we present a case of a persistent MVO-like lesion in a patient with previous radiofrequency ventricular tachycardia (VT) ablation post-myocardial infarction. Hereby, we illustrate the importance of a novel dark-blood LGE technique for detection of (chronic) ablation lesions and differentiation of MVO from other conditions.

## Clinical presentation

A 63-year-old male patient with high cardiovascular risk profile was initially hospitalized for late presentation anteroseptal myocardial infarction. Nevertheless, successful revascularization with percutaneous coronary intervention could be achieved. In the following days, several episodes of non-sustained and sustained VT occurred, requiring radiofrequency VT ablation. Hereafter, the patient was discharged without further complications and follow-up CMR was planned to evaluate left ventricular function post-myocardial infarction.

### Investigations and differential diagnosis

**Figure 1.**
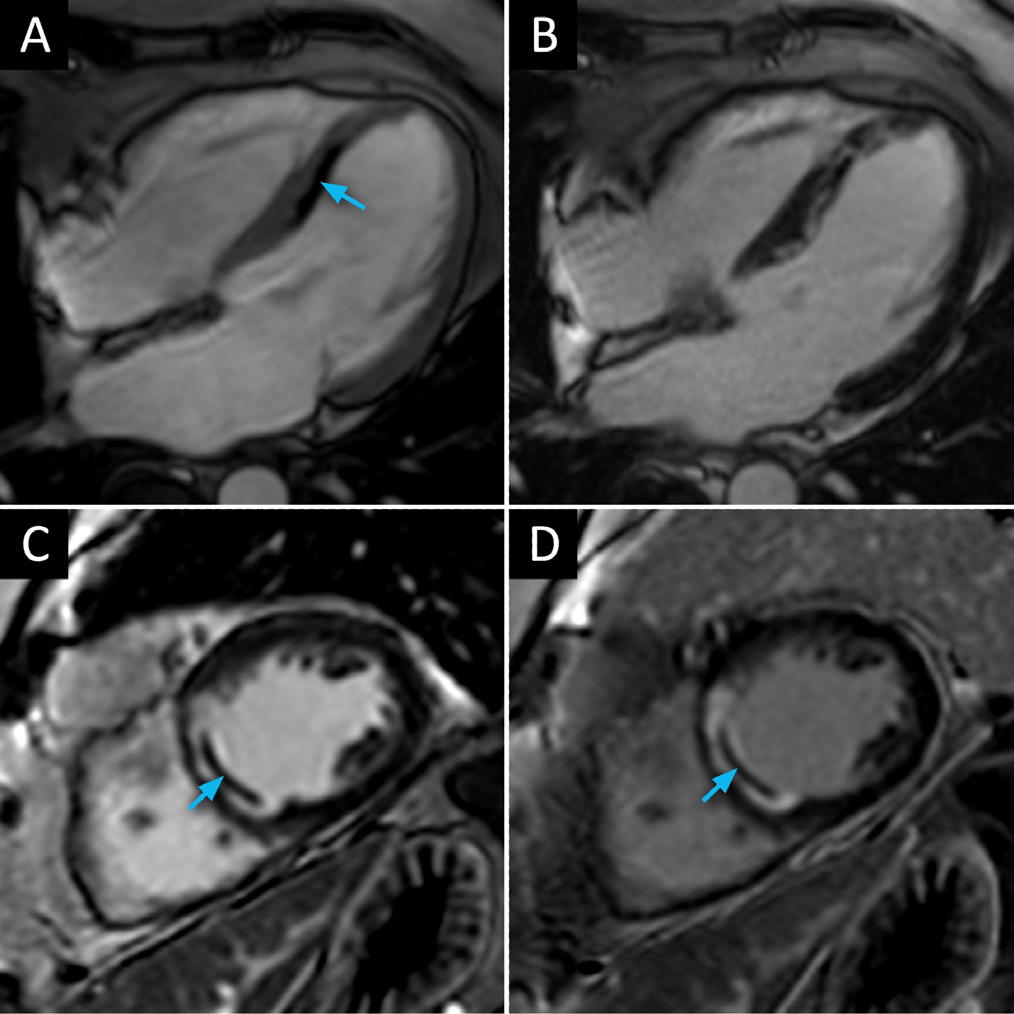
Contrast-enhanced cardiac MRI of a microvascular obstruction-like lesion (**A**) EGE image acquired in four-chamber view. (**B**) Conventional bright-blood LGE image acquired in four-chamber view. (**C**) Conventional bright-blood LGE image acquired in short-axis view. (**D**) Novel dark-blood LGE image acquired in short-axis view. The septal hypoenhancement is indicated by blue arrows. While there was no clear delineation of scar on bright-blood LGE in panel C, mimicking wall thinning and adjacent thrombus, there was an apparent ischemic scar with ‘dark core’ seen on dark-blood LGE, in keeping with a microvascular obstruction-like lesion. EGE, early gadolinium enhancement; LGE, late gadolinium enhancement.

CMR was performed 5 months after VT ablation using a clinical 3T MR scanner (Skyra; Siemens Healthineers, Erlangen, Germany). A short-axis cine stack was acquired to assess left ventricular function and post-myocardial infarction remodelling. Hypokinesia was observed at the mid-ventricular and apical segments of the septum. Global left ventricular ejection fraction was calculated at 53%. 2 min after intravenous injection of a gadolinium-based contrast agent, early gadolinium enhancement (EGE) imaging was performed which showed an area of subendocardial mid-septal hypoenhancement on the four-chamber view (Figure 1 panel A). Approximately 10 min after contrast administration, conventional and dark-blood LGE imaging were performed using a standard 2D phase sensitive inversion recovery (PSIR) LGE sequence, preceded by a standard inversion time (TI) localizer sequence. For conventional bright-blood LGE the TI was set to null the normal myocardium, while for dark-blood LGE the TI was set to null the left ventricular blood pool as previously described.^1^ All other sequence parameters were kept identical. On the four-chamber view, conventional LGE revealed a mid-septal dark area, slightly smaller compared to EGE, next to a bright zone in keeping with subendocardial ischemic scar (Figure 1 panel B). On the short-axis LGE images, no clear delineation between the bright blood-pool and potential areas of hyperenhancement could be made due to poor scar-to-blood contrast (Figure 1 panel C). As a result, the observed area of hypoenhancement appeared to be compatible with a thrombus, adjacent to an area of regional wall thinning. On the other hand, the presence of MVO could fit this finding as well. Additional dark-blood LGE was performed and demonstrated that the zone of hypoenhancement was actually a dark core, completely enclosed by a volume of myocardial scarring (Figure 1 panel D). This phenomenon could therefore be defined as an MVO-like lesion because of its location within the subendocardial scar volume. Interestingly, the MVO-like lesion corresponded to the area of radiofrequency VT ablation that was performed 5 months earlier. By expressing superior scar-to-blood contrast, dark-blood LGE proved to be the preferred option to discriminate between possible interpretations and set the correct diagnosis in this case.

## Discussion

As mentioned earlier, MVO is a phenomenon of “no reflow” observed in relation to ischemic myocardial scar formation. Using contrast-enhanced CMR sequences, it is characterized as an area with absent gadolinium enhancement within the infarct zone, and therefore appearing as a dark core within the hyperenhanced scar.^2^ In reference to the timing of imaging after contrast administration, “early” and “late” MVO can be defined.^3^ Although the appearance of MVO is broadly similar on early and late gadolinium enhancement sequences, there are important differences. Since contrast agent diffusion is impaired in the direct vicinity of MVO, it appears larger on EGE than on LGE images, as was also observed in our case. Hence, sensitivity might be higher on EGE, but LGE is currently acknowledged as the most reliable technique to detect MVO and its structural and clinical implications.^2^

The time course of MVO is known to be highly dynamic and differs depending on the clinical scenario. Following myocardial infarction, it usually appears after reperfusion and resolves after several days to weeks, reflecting areas of decreased myocardial perfusion.^2^ To the best of our knowledge, no study observed MVO at more than 2 months after acute myocardial infarction.^4^

A similar evolution has been observed with regard to MVO-like lesions post-ablation of non-infarcted areas. Several studies in patients without pre-existing myocardial fibrosis showed a transition of “dark” MVO appearance on CMR directly following ablation to “bright” LGE pattern on CMR at approximately 1 month following VT ablation.^5,6^ Chronic radiofrequency ablation lesions in regions of normal myocardium are observed to be hyperenhanced areas without the presence of MVO using LGE CMR.^6^

However, a different evolution has been observed regarding radiofrequency ablation lesions of myocardial scar in regions of previous myocardial infarction. A recent study by Dabbagh et al revealed, for the first time, the presence of a persistent MVO-like lesion on CMR in 17 patients at 30 ± 29 months after VT ablation post-myocardial infarction.^7^ Although the appearance was similar, the authors concluded that the “dark core” regions could not be diagnosed as MVO, as they were observed several years after initial myocardial infarction. Surprisingly, in that study, the differential diagnosis between the MVO-like lesions and cardiac thrombi was made by echocardiography and medical history, even though CMR is considered the most accurate technique to detect left ventricular thrombi.^8^

It can be challenging to delineate areas of hyperenhanced subendocardial scar from the bright signal of the adjacent blood pool using conventional bright-blood LGE imaging. Due to this poor scar-to-blood contrast, the full extent of myocardial scar tissue can be significantly underestimated. In our case, a novel dark-blood LGE technique was used to improve scar-to-blood contrast by creating a darker blood pool signal while preserving the dark myocardium and bright scar signals.^1^ Importantly, this dark-blood LGE technique is readily and widely available since no additional magnetization preparation mechanisms are required. Therefore, this technique can be helpful in clinical practice to improve the detection of (subendocardial) ischemic scar patterns.^9,10^ As this case illustrates, this novel LGE technique may also facilitate the discrimination of MVO from other conditions, such as left ventricular thrombus and wall thinning.

The underlying mechanisms that make these MVO-like lesions visible long after radiofrequency ablation in pre-existing myocardial ischemic scar, while this is not the case for other types of post-ablation lesions, remain unknown. The clinical history and CMR findings in our case are consistent with the recent observations of Dabbagh et al.^7^ In their study, the dark cores corresponded with unexcitable areas, and thus might represent effective ablation lesions with dense scarring. Moreover, there has been evidence that the presence of MVO post-ablation is associated with a lower incidence of VT recurrence.^11^ Although different wash-in kinetics of very dense scar might be a possible explanation for the persistence of MVO-like lesions, further studies and histological validation are required to unravel the underlying pathophysiology and evaluate clinical implications.^6^

## Conclusions

The phenomenon of persistent MVO-like lesions after VT ablation post-myocardial infarction has only recently been discovered and is supported by the findings illustrated in this case. The pathophysiology and clinical significance of these lesions in this specific setting remain unclear. Novel dark-blood LGE, compared to conventional bright-blood LGE, may be the preferred method to detect and differentiate (chronic) ablation lesions due to its superior scar-to-blood contrast.

## Learning objectives

- MVO-like lesions can persist for several months or years post-VT ablation in patients with previous ischemic scar.
- Dark-blood LGE improves differentiation of MVO and left ventricular thrombus due to its superior scar-to-blood contrast.
